# In Situ Pipe Prover Volume Measurement Method

**DOI:** 10.3390/s24154873

**Published:** 2024-07-26

**Authors:** Jiacheng Hu, Weikang Zhou, Aijun Chen, Jiale Cai, Jing Yu, Zhengzhiyong Cui, Dongsheng Li

**Affiliations:** College of Metrology and Measurement Engineering, China Jiliang University, Hangzhou 310018, China; 18897827504@163.com (W.Z.); chenaijun@cjlu.edu.cn (A.C.); a534987977@163.com (J.C.); yujing@cjlu.edu.cn (J.Y.); cuizhengzy0204@163.com (Z.C.); lidongsheng@cjlu.edu.cn (D.L.)

**Keywords:** dimensional method, geometry measurement, volume of pipe prover, measurement uncertainty

## Abstract

To improve the accuracy of in situ measurement of the standard volumes of pipe provers and to shorten the traceability chain, a new method of in situ pipe prover volume measurement was developed alongside a supporting measurement device. This method is based on the geometric dimension approach, which measures the inner diameter and length of a pipe prover to calculate its volume. For inner diameter measurement, a three-probe inner-diameter algorithm model was established. This model was calibrated using a standard ring gauge of Φ313 mm, with the parameters calculated through fitting. Another standard ring gauge of Φ320 mm was used to verify the inner diameters determined by the algorithmic model. A laser interferometer was employed for the segmented measurement of the pipe prover length. The comprehensive measurement system was then used for in situ measurement of the standard pipe prover. The newly developed system achieved an expanded uncertainty of 0.012% (*k* = 2) in volume measurement, with the deviation between the measured and nominal pipe prover volumes being merely 0.007%. These results demonstrate that the proposed in situ measurement method offers ultra-high-precision measurement capabilities.

## 1. Introduction

As essential trade-measuring instruments [1], standard pipe provers are widely used in the oil and gas industry for trade transactions. Volume deviations in pipe provers can lead to unfair trade practices and significant economic losses. Moreover, pipe provers serve as industrial calibration devices [2], crucial for the production, transportation, and storage of oil and gas. Any deviation in their volume calibration can hinder the detection of issues during these processes, such as oil leakage and seepage, potentially causing serious safety accidents and endangering lives and property. Therefore, ensuring the accurate calibration of pipe provers is of great significance.

Pipe prover measurement methods primarily include the volumetric method, gravimetric method, and master meter proving solution. The volumetric method uses a certified volumetric tank prover [3] as a standard measuring tool and calculates the volume of the pipe prover based on the measured liquid level, density, and temperature of the volumetric tank [4]. This method necessitates tracing the volumetric tank as an intermediate transfer standard, accomplished through the dimensional method [5]. In addition, the shape and appearance of the volumetric tank can affect the measurement [6], which imposes significant limitations on the volumetric method. 

The gravimetric method involves filling the pipe prover with an intermediate medium, discharging the medium into a gravimetric tank, and then weighing the medium. This method uses the weight of the medium to convert the volume of the pipe prover, utilising standard scales and weights as standard measuring tools.

In 2016, Doihara used a gravimetric tank and piston to measure the volume of a pipe prover, achieving an expanded uncertainty of 0.066% (*k* = 2) [7]. However, this method imposes strict requirements on the calibration process. During measurement, pressure and temperature can vary rapidly and unevenly, while the viscosity and density of the medium significantly affect measurement accuracy [8]. Consequently, correcting the measurement results is crucial [9]. The master meter proving solution employs a standard flow meter as the transfer standard between the pipe prover and the measuring instrument, using flow parameters to indirectly measure the pipe prover volume. In 2015, Shimada verified the volume of a pipe over 15 m long with an inner diameter of 150 mm using the master meter method. The expanded uncertainty of the volume flow rate reached 0.03%, and the uncertainty of the mass flow rate reached 0.02% (*k* = 2) [10]. In the same year, the National Metrology Institute of the Netherlands used a standard flow meter to calibrate a 500 L volume tank, achieving an expanded volume measurement uncertainty of less than 0.04% (*k* = 2). However, verifying the stability and continuity of a standard flow meter through actual measurements is challenging, making cumulative errors difficult to avoid [11].

After analysing the uncertainty of the volume tube used in the volumetric method, Lim [12] and Oracheski [13] believed that the standard volume tank itself played a dominant role. This greatly limits the further improvement of the volumetric measurement accuracy. When the mass method measures small volumes, it is limited by the principle of mass measurement, and the measurement accuracy is difficult to improve further. When Toshihiro Morioka and others used the standard flow meter method for measurement, the relative measurement uncertainty of the standard flow meter itself reached 0.09%, and in the case of large fluctuations [14], the relative measurement uncertainty reached 0.44%. In addition, the standard flow meter can usually obtain good measurement accuracy in the low flow area, but it is difficult to obtain high accuracy in the high flow area [15]. These methods indirectly measure the pipe prover volume through intermediate media, which inevitably extends the traceability chain to the SI unit [16]. The length of the traceability chain directly impacts measurement accuracy, significantly limiting the efficacy of these methods. Therefore, reducing the traceability chain length in pipe prover volume measurement is crucial for improving accuracy.

The dimensional method is an in situ approach that calculates the pipe prover volume by measuring the length and inner diameter of a standard pipe prover segment. This method can be directly traced to the length benchmark, significantly improving measurement accuracy. In 2003, Többen developed an axial and radial incremental length measurement device and used it to measure the geometric quantity of a pipe prover with a nominal volume of 250 L. The measured volume was compared with that obtained using the gravimetric method, with a difference of only 0.008% [17]. Többen was the first to use the dimensional method to measure a pipe prover, demonstrating the potential of this method for a more accurate characterisation of pipe prover volumes. Compared with traditional measurement methods, this method is less susceptible to external factors and has looser environmental requirements. With the continuous improvement of geometric measurement accuracy, the dimensional method has more advantages in measuring the volume of a pipe prover.

The feasibility of using the dimensional method to measure pipe provers has been verified in previous studies. However, when using dual probes to measure the inner diameter, possible installation deviations of the sensor, such as the installation angle and eccentric distance, have been ignored. The influence of factors such as the eccentric distance and the lack of measurement uncertainty analysis in this method make verifying the reliability of this measurement method challenging. Therefore, this paper proposes a new method for pipe prover volume detection. In this approach, three probes are used to measure the inner diameter. An inner diameter algorithm model is utilised that includes installation angle, eccentric distance, and measured arm length parameters. The length is measured using a laser interferometer. A supporting measurement device is employed to complete the in situ measurement and uncertainty analysis of the pipe prover volume.

## 2. Measurement Method

**Figure 1:**
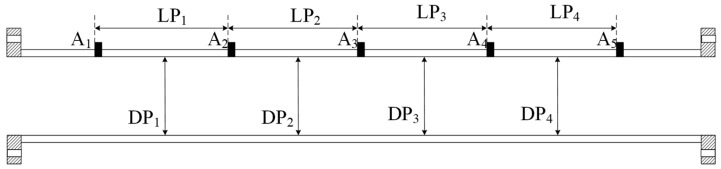
Pipe prover volume calculation method.

A pipe prover is a hollow cylinder measuring instrument with a segmented design. In this study, the pipe prover was divided into multiple standard segments using a detection switch. Consequently, the pipe prover volume measurement is based on the addition of the volumes of multiple segments, as shown in Equation (1):

(1) $$\left\{\begin{array}{l} V_{i}=\pi \times {\left(\frac{{\mathrm{DP}}_{i}}{2}\right)}^{2}\times {\mathrm{LP}}_{i}\\ V=\sum_{i=1}^{4}V_{i} \end{array}\right.\left(i=1,2,3,4\right),\;$$

where, DP*_i_*, LP*_i_,* and *V_i_* represent the inner diameter, length, and volume of each standard segment of the pipe prover, respectively, and *V* is the total volume of the standard segments of the pipe prover. The calculation principle for the pipe prover volume is illustrated in Figure 1. The inner diameter and length of the pipe prover must be measured separately. High-precision measurements of the inner diameter of a pipeline are typically performed by measuring the relative displacement of the sensor [18]. This method cannot directly obtain the inner diameter of the measured object; therefore, an inner diameter algorithm model must be established, followed by using the relative measurement value of the sensor to calculate the inner diameter of the pipe. Segmented length measurements were required in this study because the pipe prover had a segmented design.

### 2.1. Inner Diameter Measurement Method

**Figure 2:**
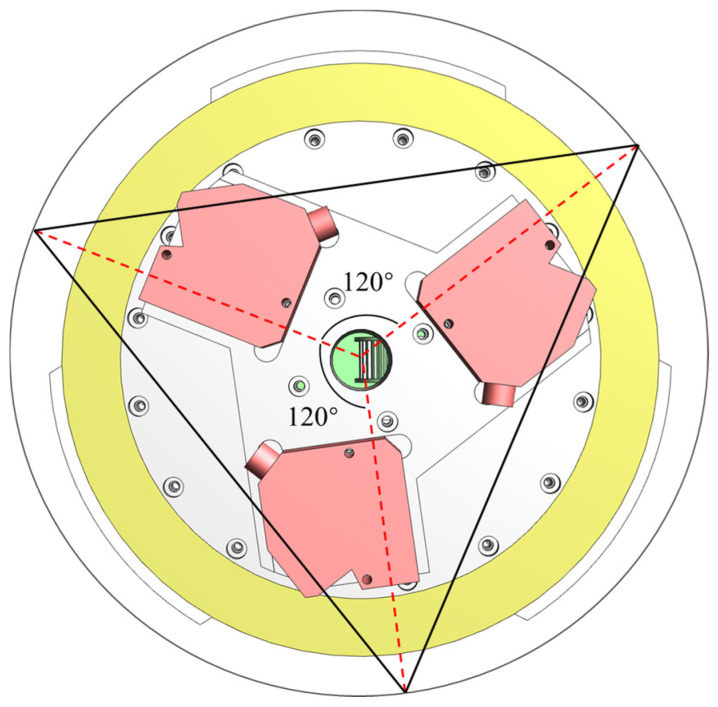
Principle of three-point circle determination.

The inner diameter measurement method was based on the three-point circle principle [19], as shown in Figure 2. Under ideal conditions, three coplanar laser displacement sensors installed at angles of 120° with respect to one another are used to measure the distance from the wall of the pipe prover. The distances between the measurement points of sensors form a triangle, and the radius of its circumscribed circle is determined.

**Figure 3:**
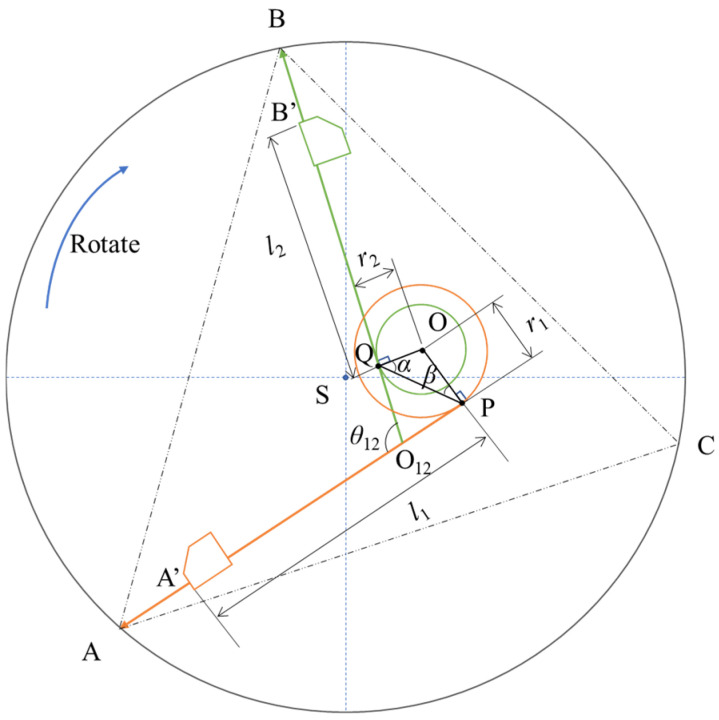
Principle of three-probe inner diameter algorithm model.

However, in an actual measurement environment, deviations in the installation angles of the three laser displacement sensors will exist, meaning the angles between the sensors are not precisely 120°. Considering that the actual rotation centre of the three sensors will differ from that of the measured pipe prover, the centres of the circles do not coincide, causing a deviation between the axis of each sensor and the centre of the circle. The three-probe inner-diameter algorithm model based on this scenario is shown in Figure 3.

In Figure 3, *A′* and *B′* are the measurement starting points of the laser displacement sensors, *S* is the centre of the circle of the measured pipe prover, *O* is the actual rotation centre of the inner diameter measurement module, *O*_12_ is the intersection point of measurement beams *AA′* and *BB′*, and *θ*_12_ is the angle between the measurement beams. During rotation measurement, taking measurement beams *AA′* and *BB′* as examples, each beam rotates around point *O*, forming two concentric tangent circles of the beam. *OQ* and *OP* are the radii of the tangent circles, denoted as *r*_1_ and *r*_2_, respectively. *A′P* and *B′Q* are the distances from the measurement starting point to the installation starting point, denoted as *l*_1_ and *l*_2_, respectively. *α* and *β* are the angles for auxiliary calculation, expressed as *α* = ∠*OQP* and *β* = ∠*OPQ*, respectively.

In the three-probe inner diameter algorithm model, the unique triangle *ABC* formed by the measurement points can be obtained. The side length *AB* in the triangle can be calculated using the cosine theorem:

(2) $$AB=\sqrt{O_{12}A^{2}+O_{12}B^{2}-2\cdot O_{12}A\cdot O_{12}B\cdot \cos\theta_{12}}.$$

*O*_12_*A* and *O*_12_*B* can be expressed as

(3) $$\left\{\begin{array}{l} O_{12}A=l_{1}+AA\prime -O_{12}P\\ O_{12}B=l_{2}+BB\prime +O_{12}Q \end{array}\right.,\;$$

where *AA′* and *BB′* are the measured values of the sensor and *l*_1_ and *l*_2_ are the lengths of the measuring arms. Therefore, *AB* can be calculated by determining *O*_12_*P* and *O*_12_*Q*.

In triangle *OPQ*, it can be known from the sine formula:

(4) $$\frac{\sin\alpha }{r_{2}}=\frac{\sin\beta }{r_{1}}=\frac{\sin\theta_{12}}{PQ}.\;$$

*α* and *β* in Equation (4) can be obtained as

(5) $$\left\{\begin{array}{l} \beta =\sin^{-1}\left(r_{1}\frac{\sin\theta_{12}}{PQ}\right)\\ \alpha =\sin^{-1}\left(r_{2}\frac{\sin\theta_{12}}{PQ}\right) \end{array}\right..\;$$

In the quadrilateral *OQO*_12_*P*, *PQ* is also obtained from the cosine formula:

(6) $$PQ=\sqrt{{r_{1}}^{2}+{r_{2}}^{2}-2\cdot r_{1}\cdot r_{2}\cdot \cos\theta_{12}},\;$$

and in triangle *O*_12_*PQ*, it can be found by using the sine formula:

(7) $$\frac{\sin(\frac{\pi }{2}-\alpha )}{O_{12}P}=\frac{\sin(\frac{\pi }{2}-\beta )}{O_{12}Q}=\frac{\sin(\pi -\theta_{12})}{PQ}.\;$$

*O*_12_*P* and *O*_12_*Q* in Equation (7) can be obtained by applying

(8) $$\left\{\begin{array}{c} O_{12}Q=PQ\frac{\sin\left(\frac{\pi }{2}-\alpha \right)}{\sin\left(\pi -\theta_{12}\right)},\\ O_{12}P=PQ\frac{\sin\left(\frac{\pi }{2}-\beta \right)}{\sin\left(\pi -\theta_{12}\right)}. \end{array}\right.$$

By substituting Equations (5) and (6) into Equation (7), *O*_12_*P* and *O*_12_*Q* can be calculated. Side length *AB* can be calculated using Equations (2) and (3), and similarly for side lengths *BC* and *CA*.

*R* can be obtained from the formula for the radius of the circumscribed circle of a triangle as follows:

(9) $$\left\{\begin{array}{l} P=\frac{AB+BC+CA}{2}\\ AB=\sqrt{O_{12}A^{2}+O_{12}B^{2}-2\cdot O_{12}A\cdot O_{12}B\cdot \cos\theta_{12}}\\ BC=\sqrt{O_{23}B^{2}+O_{23}C^{2}-2\cdot O_{23}B\cdot O_{23}C\cdot \cos\theta_{23}}\\ CA=\sqrt{O_{31}C^{2}+O_{31}A^{2}-2\cdot O_{31}C\cdot O_{31}A\cdot \cos\theta_{31}}\\ R=\frac{AB\cdot BC\cdot CA}{4\sqrt{P(P-AB)(P-BC)(P-CA)}}\\ D=2R \end{array}\right.$$

*AB*, *BC*, and *CA* are calculated by *AA′*, *BB′*, *CC′*, *θ*_12_, *θ*_23_, *θ_3_*_1_, *l*_1_, *l*_2_, *l*_3_, *r*_1_, *r*_2_, and *r*_3_. The inner diameter *D* of the smallest circumscribed circle of triangle ABC can be expressed as

(10) $$D=f\left(l_{1},l_{2},l_{3},r_{1},r_{2},r_{3},\theta_{12},\theta_{23},\theta_{31},AA^{\prime },BB^{\prime },CC^{\prime }\right)$$

The measured pipe volume was divided into four segments in this study. When measuring the first segment, the inner diameter calculated using the three-probe inner diameter algorithm model represented the inner diameter of a single sampling point in a single section. To accurately reflect the inner diameter of a single section *n* sampling measurements were conducted to obtain *D*_11*j*_ (*j* = 1, 2, 3, …, *n*). However, the inner diameter of a single section cannot accurately represent the inner diameter of a pipe segment. Therefore, *m* cross-sectional measurements were performed on this pipe segment, resulting in *D*_1*ij*_ (*i* = 1, 2, 3, …, *m*; *j* = 1, 2, 3, …, *n*). The average of these sampled measurements, *D*_1_, was used as the inner diameter of the first segment of the pipe prover:

(11) $$D_{1}=\sum_{i,j=1}^{m,n}\frac{1}{n\times m}D_{1,i,j}(i=1,2,3,\cdots ,m;j=1,2,3,\cdots n)$$

The inner diameters *D*_2_, *D*_3_, and *D*_4_ of the remaining three pipe segments were calculated using the same method.

### 2.2. Length Measurement Method

The length of the pipe prover was measured using a laser interferometer. Detection switches were present at both ends of each segment of the pipe prover. A trigger was installed on the measurement device to activate the detection switch. When the first segment was measured, detection was triggered, and the reading of the laser interferometer was recorded. The difference in the readings of the laser interferometer at both ends of the segment, activated by the detection switch, was taken as the length *L*_1_ of the first segment of the pipe prover:

(12) $$L_{1}=\left|L_{11}-L_{12}\right|.\;$$

*L*_2_, *L*_3_, and *L*_4_ of the remaining three pipe segments of the volume pipe were calculated using the same method.

## 3. Experimental Setup

**Figure 4:**
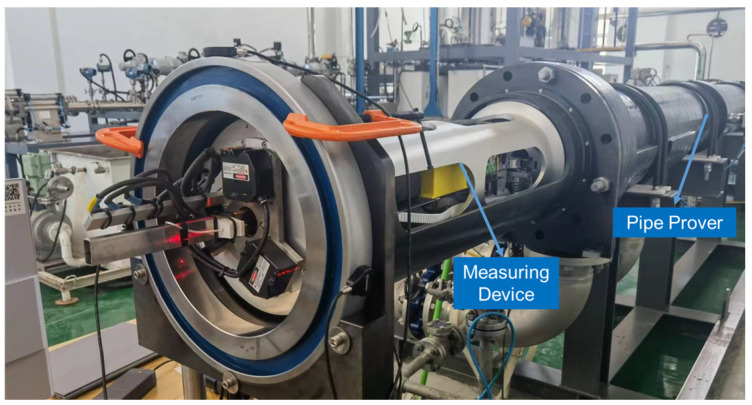
Experimental setup.

**Figure 5:**
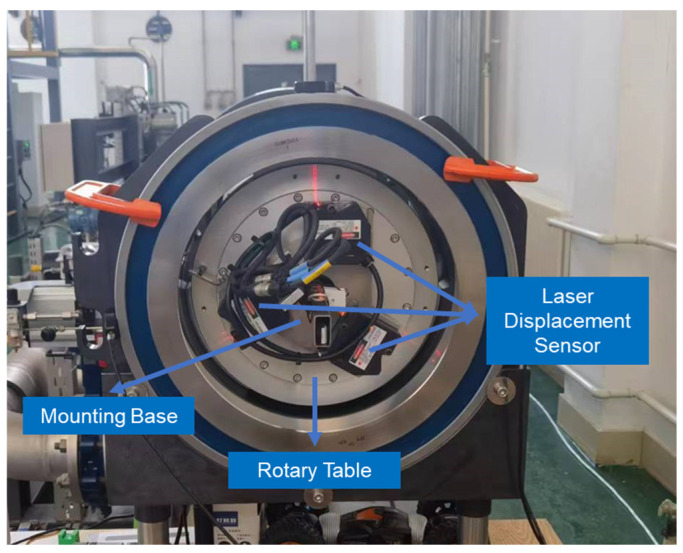
Diameter measurement module.

**Figure 6:**
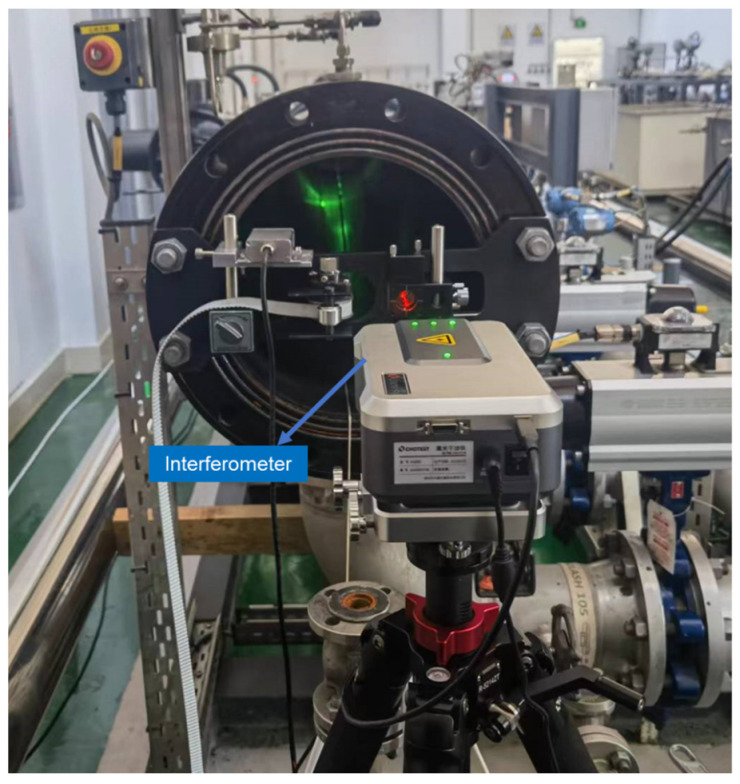
Length measurement module.

**Figure 7:**
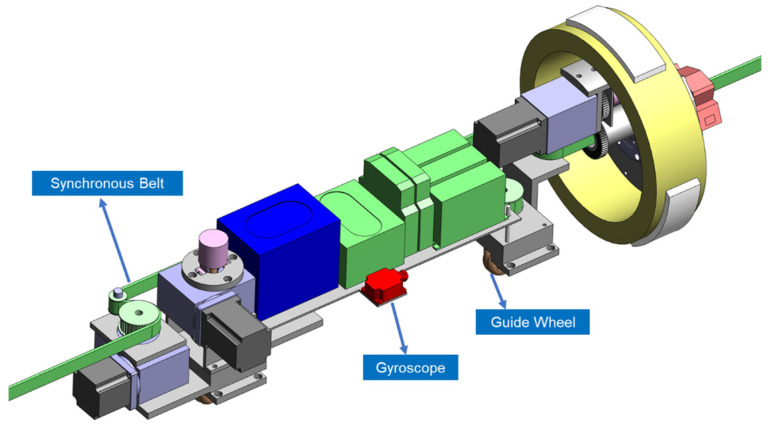
Motion and posture adjustment module.

The experimental device, shown in Figure 4 consists of an inner diameter measurement module (Figure 5), a length measurement module (Figure 6), and a motion control module (Figure 7).

### 3.1. Inner Diameter Measurement Module

The inner diameter measurement module, developed based on the three-point circle principle, is shown in Figure 5. This module mainly comprises a rotating stage and three laser displacement sensors. The three laser displacement sensors are installed at angles of 120° relative to one another. Each sensor is positioned equidistantly from the centre of rotation and fixed on the mounting base, which rotates with the rotary table. The entire inner diameter measurement module moves within the pipe prover along with the measurement device, enabling the measurement of different sections of the pipe prover.

The laser displacement sensor used to measure the inner diameter is a KEYENCE sensor, with a measurement accuracy of 2 μm and a measurement range of ±10 mm, which meets the requirements for inner diameter measurement accuracy and range. To improve the accuracy of the inner diameter measurement, an electric rotary table from IKO is utilised, with both axial and radial runout less than 5 μm.

### 3.2. Length Measurement Module

The length measurement module is shown in Figure 6. This module primarily consists of a laser interferometer fixed at one end of the pipe prover, while the reflector is attached to the measurement device. The measurement device triggers the detection switch between each pipe segment, synchronously recording data from the laser interferometer and completing the length measurement of each pipe segment through the device’s movement.

The laser interferometer is a CHOTEST single-frequency interferometer with a length measurement accuracy of up to 0.5 ppm, accommodating a length measurement range of up to 40 m. To ensure stable movement of the laser interferometer mirror and maintain a continuous and uninterrupted light path, we specially designed PTFE [20] blocks surrounding both sides of the measurement device. Springs are installed inside the PTFE blocks to expand outward, achieving a fit between the measurement device and the inner wall of the pipe. This setup ensures precise and accurate in situ measurements of both the inner diameter and length of the pipe prover segments, significantly enhancing the reliability of the overall volume measurement.

### 3.3. Motion Control Module

The motion control module, shown in Figure 7, primarily comprises a synchronous belt, gyroscope, and guide wheel. The synchronous belts are fixed at both ends of the pipe prover, allowing the measurement device to move along the belt.

The gyroscope is a WitMotion attitude sensor, with an inclination accuracy of 0.001° and a measurement range of ±90°, meeting the operational requirements. To avoid the effects of pitch and yaw angles on the inner diameter and length measurement results during movement, the gyroscope continuously reads the attitude information of the measurement device. This allows the motion control module to control the device in real time.

## 4. Experiments and Results

### 4.1. Experimental Pipe Prover

A standard pipe prover was selected as the measurement object, divided into four standard segments: P_1_, P_2_, P_3_, and P_4_. The nominal volumes of these segments, based on the design indicators of the pipe prover, are listed in Table 1.

**Figure 8:**
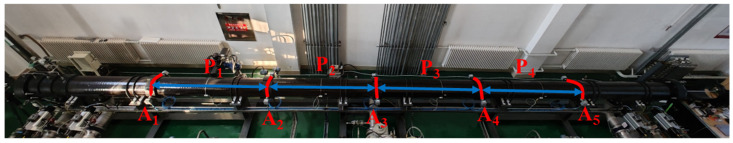
Standard pipe prover.

The pipe prover to be measured is made of glass-fibre-reinforced epoxy resin material, with a total length of approximately 12 m. Two buffer segments, approximately 1 m long each, are present at both ends of the pipe prover, with a standard pipe segment approximately 8 m long in the middle. As shown in Figure 8, detection switches A_1_, A_2_, A_3_, A_4_, and A_5_ are located at the beginning and end of pipe segments P_1_, P_2_, P_3_, and P_4_.

The experimental site has the ability to regulate temperature. During the entire experiment, the temperature range of the volume tube and the measuring device is 20 ± 2 °C.

### 4.2. Calibration of Inner Diameter Three-Probe Algorithm Model 

According to the three-probe inner diameter algorithm model described by Equation (10), the inner diameter of the measured section was calculated using 12 parameters. However, in the actual measurement, only three of these parameters—AA′, BB′, and CC′ of the laser displacement sensor—are measured. These parameters can be obtained through sensor readings and vary with the measurement position, while the remaining nine parameters remain constant after the device is installed. 

Due to the use of a non-contact measurement method involving the laser displacement sensor, measuring the angle between the optical path of the laser displacement sensor and the origin is challenging. Consequently, angles *θ*_12_, *θ*_23_, and *θ*_31_ as well as offsets *r*_1_, *r*_2_, and *r*_3_ can only be determined by fitting the inverse solution. The lengths of the measuring arms along with a known minimum circumscribed circle inner diameter *D* of the triangle, must also be provided for Equation (10), so that *θ*_12_, *θ*_23_, and *θ*_31_ and *r*_1_, *r*_2_, and *r*_3_ can be solved through fitting. 

Since the number of unknown parameters affects the accuracy of the fitted inverse solution [21], an increase in unknown parameters reduces the degree of fitting. After comprehensive consideration, the included angle and offset distance [22] which have greater impacts on the measurement results, were selected as the unknown parameters.

**Figure 9:**
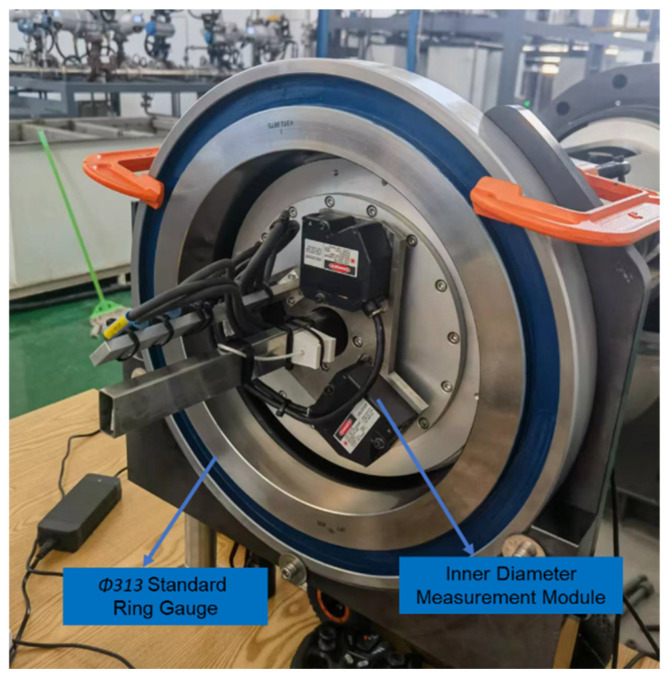
Φ313 mm standard ring gauge.

Two standard ring gauges composed of bearing steel were machined as standard values for the inner diameter algorithm model. The nominal sizes of these gauges were Φ313 mm and Φ320 mm. The Φ313 mm standard ring gauge is shown in Figure 9.

The parameters of the Φ313 mm standard ring gauge were obtained after metrological verification and are listed in Table 2.

**Figure 10:**
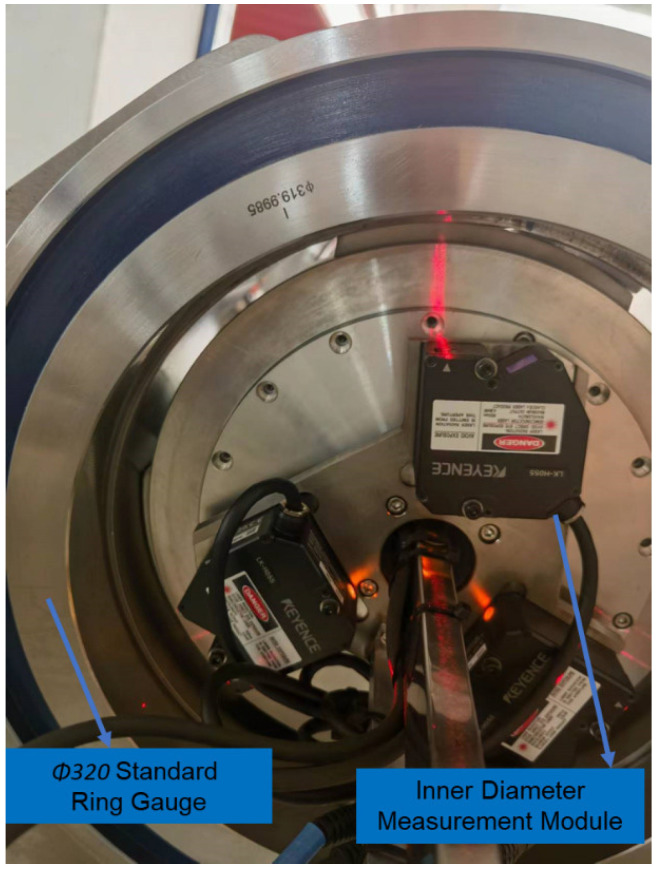
Φ320 mm standard ring gauge.

The ring gauge with a nominal size of Φ320 mm is shown in Figure 10.

The parameters of the Φ320 mm standard ring gauge were obtained after metrological verification and are listed in Table 3.

**Figure 11:**
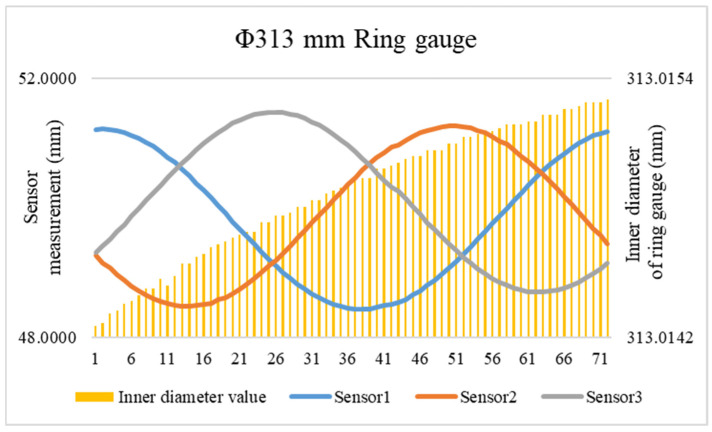
Data used for calibration.

The nominal value of the standard ring gauge is the standard length at a temperature of 20 °C. When using the standard ring gauge for calibration, temperature compensation must be performed on the inner diameter length of the standard ring gauge [23]. Considering that the material used in the standard ring gauge is bearing steel, the linear expansion coefficient *α_R_* is 14 × 10^−6^/°C, and the mounting base of the laser displacement sensor is made of Invar alloy, with a linear expansion coefficient *α_MD_* of 0.8 × 10^−6^/°C. When calibrating using the Φ313 mm standard ring gauge, the measurement device is in operation, causing the device to heat up slowly, which affects the temperature of both the standard ring gauge and the mounting base. During this period, the data were temperature-compensated to obtain the measured values of the three laser displacement sensors and the nominal values of the standard ring gauge. When the sampling angle interval between the sensors and the standard ring gauge was 5°, each sensor measured 72 data points after one rotation. The obtained data are presented in Figure 11.

Using this set of data for the fitting calculation of the inner diameter algorithm model, the values of each parameter of the inner diameter algorithm model were calculated and the fitting values were temperature-compensated. The parameter results are listed in Table 4.

**Figure 12:**
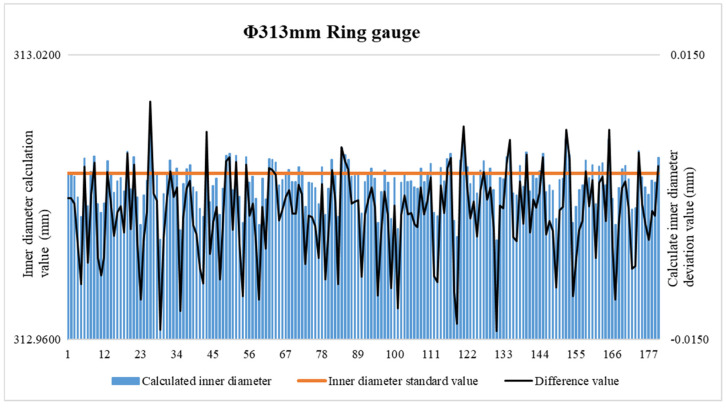
Φ313 mm ring gauge data used for verification.

As the parameters of the inner diameter algorithm model are obtained through fitting and solving, certain errors must exist in the fitting values [24]. Therefore, the fitting effect of each parameter needs to be verified. For this purpose, a ring gauge (nominally Φ313 mm under the 20 °C standard condition) was used. The inner diameter of the standard ring gauge and that obtained from the inner diameter algorithm model were compared, as shown in Figure 12.

**Figure 13:**
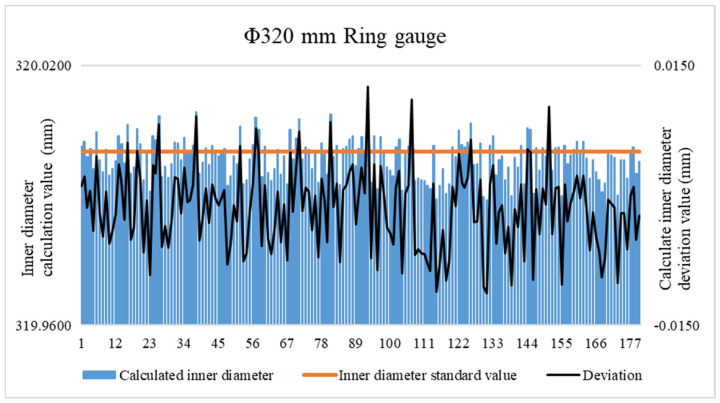
Data used for verification.

The quality of the fitting effect could not be verified using a single ring gauge. Additional data were required for verification [25]. For this purpose, a ring gauge (nominally Φ320 mm under the 20 °C standard condition) was used. The inner diameter of the standard ring gauge and that obtained from the inner diameter algorithm model were compared, as shown in Figure 13. The deviation between the average value of this data set and the standard value of the inner diameter is 1.50 μm, and the standard deviation is 1.37 μm.

### 4.3. Inner Diameter Measurement Results

**Figure 14:**
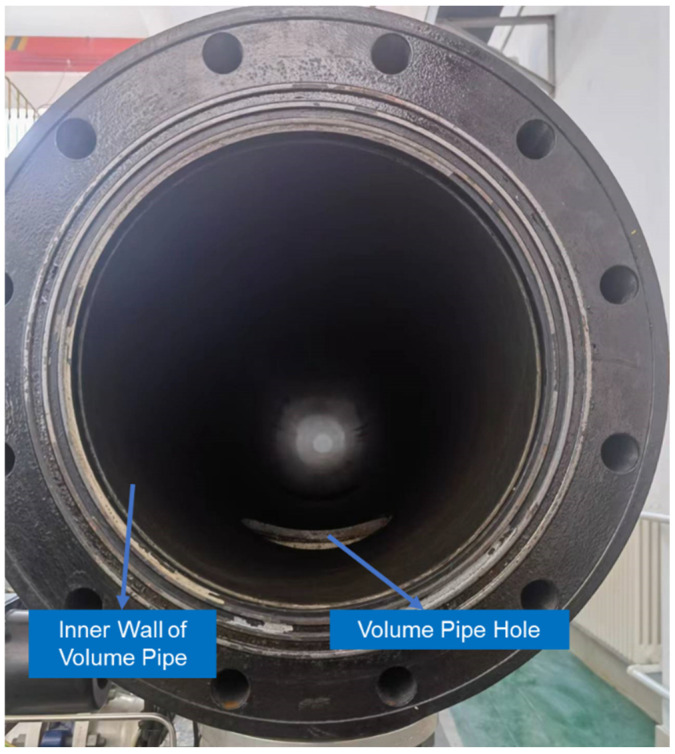
Inner wall of volume pipe.

The pipe prover was measured in a 20 ± 2 °C environment, as shown in Figure 14.

During the measurements, the measurement device moved to standard pipe segment P_1_ of the pipe prover and began measuring at the beginning of this segment. When measuring the first section, samples were taken at 20° intervals and repeated three times to complete the measurement of one section. The measurement device was then moved forward by 100 mm to measure the second section of the standard pipe segment, and the above steps were repeated until the inner diameter measurement of the standard pipe segment was completed. During the inner diameter measurement process, stabilization was performed for 5 s after each rotation of the sampling point to reduce the impacts of the axial and radial runouts of the turntable on the inner diameter measurement results. 

The pipe prover is made of glass-fibre-reinforced epoxy resin, with a radial linear expansion coefficient *α*_PD_ of 34.6 × 10^−6^/°C. The inner diameter of the pipe prover was temperature-compensated. The cross-sectional inner diameters obtained throughout the measurement process are presented in Table 5.

### 4.4. Length Measurement Results

The measurement device simultaneously measured the length of the inner diameter and the standard segment of the pipe prover in segments, and repeated the measurement on the detection switch of each segment 10 times. The axial linear expansion coefficient *α_PL_* of the pipe prover is 17.3 × 10^−6^/°C. The pipe prover length measurement results were temperature-compensated. The lengths of the pipe segments are listed in Table 6.

### 4.5. Volume Calculation Results

The volume of the pipe prover was calculated using the length and inner diameter of each segment, and the results are presented in Table 7.

According to Table 1, the standard volume V_STD_ of the pipe prover is 602.78 dm^3^, whereas the volume V_MEA_ calculated after measuring the length and inner diameter of the pipe prover using the measurement device is 602.74 dm^3^. Hence, the deviation V_ERR_ is 0.04 dm^3^, the relative deviation V_RSD_ can be calculated to be 0.0066% by applying Equation (13):

(13) $$V_{\mathrm{RSD}}=\frac{V_{\mathrm{MEA}}-V_{\mathrm{STD}}}{V_{\mathrm{STD}}}\times 100\%=\frac{V_{\mathrm{ERR}}}{V_{\mathrm{STD}}}\times 100\%.\;$$

## 5. Uncertainty Analysis

The uncertainty components for volumetric tube volume measurement can be described as follows.

(1) The uncertainty components of the inner diameter measurement include the (a) repeatability of the inner diameter measurement results, (b) indication error of the inner diameter measurement, (c) temperature compensation error of the standard ring gauge, (d) standard ring gauge linear expansion coefficient error, (e) standard ring gauge traceability, (f) inner diameter error of the measurement results caused by pipe roundness, (g) pipe prover radial temperature compensation error, (h) pipe prover radial linear expansion coefficient error, (i) pipe prover pressure deformation error, and (j) pipe prover hydraulic pressure deformation error.

(2) The uncertainty components of the length measurement include the (a) repeatability of the length measurement results, (b) laser interferometer measurement error, (c) signal delay error, (d) axial temperature change error, and (e) pipe prover axial linear expansion coefficient error.

### 5.1. Uncertainty Analysis of Inner Diameter Measurement

The inner diameter measurement results of multiple measurements of a single section of the volume tube are shown in Table 8. The standard deviation of the inner diameter measurement results is calculated to be 4.06 μm.

**Figure 15:**
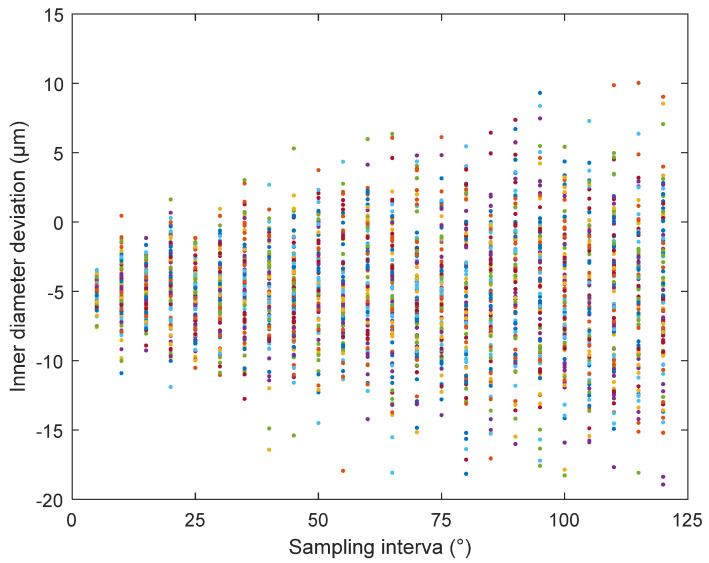
Influence of roundness on measurement.

Because the inner diameter algorithm model utilises a three-point circle method, certain errors can occur when measuring the cross-section of a non-standard circle [26]. After assessing the full roundness of the standard segment of the pipe prover, the maximum roundness deviation was found to be 61.87 μm. To analyse the impact of this roundness deviation, a computer simulation was set up with a simulated circle having a roundness of 61.87 μm. Different measurement sampling intervals were tested, with simulations performed in 5° steps from 1° to 120°, covering a full 360° sampling of the simulated circle. The calculated inner diameter of each sampling point was obtained after inputting the values into the algorithm model [27]. The average inner diameter of the simulated circle was then calculated, and the difference from the standard circle was noted. This process was repeated 100 times, each time setting a new roundness for the 61.87 μm simulation circle. The inner diameter deviations with respect to the sampling angle when measuring the simulation circle with a roundness of 61.87 μm are presented in Figure 15.

The simulation results indicated that smaller sampling intervals led to a lower degree of inner diameter deviation but increased the measurement time. To balance measurement accuracy and efficiency, the sampling interval during the inner diameter measurement was set to 20°. Using a 95% confidence interval for the 20° sampling data set, the maximum deviation of this data set was 5.87 μm.

**Figure 16:**
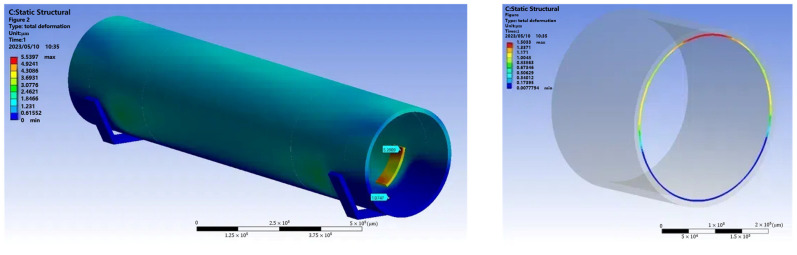
Cross-section deformation simulation.

As the measurement device puts pressure on the tube wall when it is inside the pipe prover, the deformation of the tube wall under pressure must be considered. The simulation model was established and static analysis was performed based on the actual state of the measuring device when measuring the pipe prover. During the analysis, the parameters of the volume tube and blocks were set according to the mechanical properties of glass-fibre-reinforced epoxy resin material and PTFE, and the pressure was set according to the weight of the measuring device. The deformation of the pipe prover at the measurement section was evaluated, as shown in Figure 16. The error of the inner diameter measurement module caused by compression deformation was found to be 0.8 μm.

**Figure 17:**
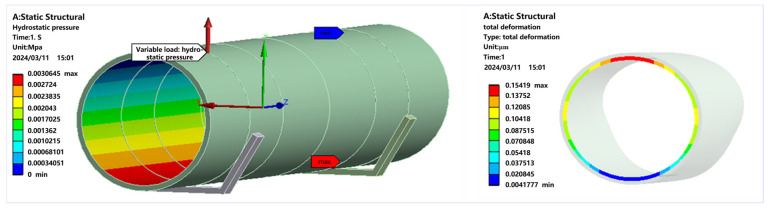
Cross-section hydraulic deformation simulation.

Furthermore, during measurement, the pipe prover is empty, but in actual use, it is filled with liquid, creating a different measurement environment. Set the internal pressure of the volume tube according to the density of the medium when the pipe prover is actually used. The full-load hydraulic deformation of the pipe prover was simulated as shown in Figure 17, revealing a hydraulic deformation error of 0.2 μm.

Throughout the measurement process, the environmental temperature fluctuated within a range of 2 °C. The sensor used for temperature compensation had an accuracy of ±0.1 °C. Considering that the wall thickness of the pipe prover was 40 mm, the thickness of the standard ring gauge was 137 mm, the linear expansion coefficient of the material had an uncertainty of 20%, and the errors were uniformly distributed, the uncertainty in the inner diameter measurement can be calculated, as listed in Table 9.

All of the uncertainty sources in Table 9 are considered to be independent of each other; therefore, the synthetic uncertainty *u*_D_ of the inner diameter measurement can be calculated as

(14) $$u_{D}=\sqrt{\sum_{i=1}^{10}{u_{Di}}^{2}}=4.25\;\mu m.\;$$

### 5.2. Uncertainty Analysis of Length Measurement 

**Figure 18:**
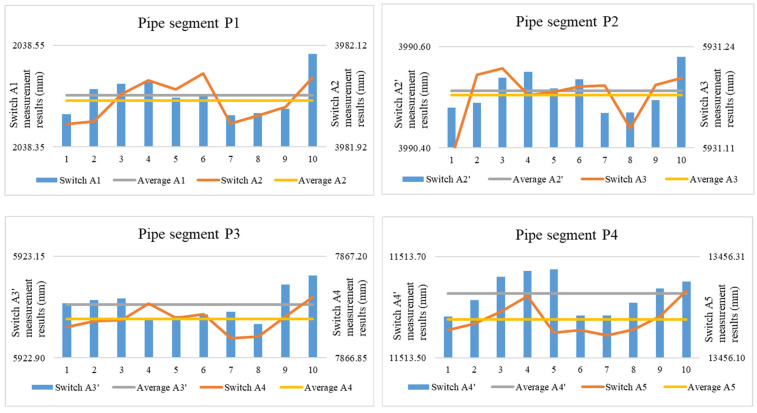
Position of pipe segment switches.

The length measurement of the pipe prover was determined by the detection switch positions along the axial direction, as shown in Figure 8. Therefore, the measured length of each pipe prover segment was the difference between the positions of the two detection switches, which can also be obtained using Equation (12). The repeatability of the pipe prover length is characterized by the repeatability of the two detection switches. The measured positions of the detection switches for each segment of the pipe prover are shown in Figure 18.

From this information, the repeatability of the detection switch of each pipe segment, repeatability of the volume pipe length measurement, and repeatability of the length measurement of each pipe segment can be obtained, as listed in Table 10.

The laser interferometer had an error indication. The expression of the indication error obtained after metrological verification is (0.03 + 10^−6^ *L*) μm, where *L* is the measurement length in meters. The indication error is related to the measurement range *L*, so the indication error of each pipe segment is different.

**Figure 19:**
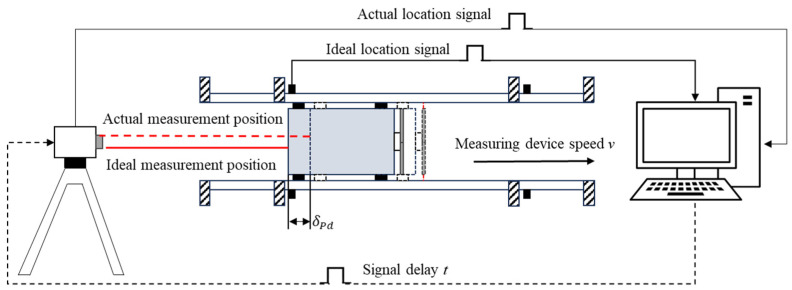
Principle of signal delay.

When the device performs a length measurement, it must move to the detection switch position to trigger the switch and record the position at that moment. The measurement process is shown in Figure 19. However, due to a time delay (*t* = 1 ms) caused by the transmission of the trigger signal from the detection switch on the pipe prover to the measurement device [28], and the measurement device moving at a speed *v*, an error occurs. When approaching the detection switch, the speed was set to *v* = 1 mm/s. This setting caused the laser interferometer to read the distance of the reflector located on the measurement device when the device was no longer at the triggering position of the detection switch. The error due to the time delay is equal to the product of this position difference and the speed.

Throughout the measurement process, the ambient temperature fluctuated within 2 °C, and the sensor accuracy for temperature compensation was ±0.1 °C. Considering that the length of each segment of the pipe prover is as shown in Table 6 and the axial linear expansion coefficient of the pipe prover material is considered to have a 20% uncertainty, the uncertainty of the length measurement can be calculated as shown in Table 11.

The length uncertainty of each pipe segment can be calculated separately by applying Equation (15):

(15) $$u_{LP_{j}}(j=1,2,3,4)=\sqrt{\sum_{i=1}^{5}u_{Li}{\;}^{2}}.\;$$

The length uncertainties for each pipe segment, *u*_LP1_, *u*_LP2_, *u*_LP3_, and *u*_LP4_, are listed in Table 12.

### 5.3. Pipe Prover Volume Uncertainty

From Equation (1) we can obtain the calculation formula for the pipe prover volume and then calculate the sensitivity coefficient formulas for the inner diameter and length, as shown in Equation (16):

(16) $$\left\{\begin{array}{l} c_{LP_{i}}=\frac{\partial V_{i}}{\partial LP_{i}}=\frac{\pi }{4}\times DP_{i}(i=1,2,3,4)\\ c_{DP_{i}}=\frac{\partial V_{i}}{\partial DP_{i}}=\frac{\pi }{2}\times DP_{i}\times LP_{i}(i=1,2,3,4) \end{array}\right.$$

According to Table 5 and Table 6, the sensitivity coefficients of each pipe segment can be calculated and are listed in Table 13.

Then, combining Equation (1) with Table 1, the relative uncertainty of volume expansion (*k* = 2) can be found to be 0.012%.

## 6. Conclusions

In this study, we measured the volume of a pipe prover in situ using a dimensional method, which required determining the length and inner diameter of the pipe prover. We developed an algorithm model for the inner diameter measurement that was suitable for scenarios involving three measuring probes. This algorithm model was calibrated and verified using two standard ring gauges with different inner diameters, demonstrating its suitability for non-contact measurements. While parameters such as the angle, offset distance, and arm length of the inner diameter measurement module were challenging to measure directly, the experiment validated the accuracy of the method. The length measurements were completed using a laser interferometer, which enabled segmented measurements. The developed measurement device is compact and capable of comprehensive geometric measurements of the pipe prover. The difference between the measured and nominal pipe prover volumes was only 0.0066%. In future work, measuring the linear expansion coefficient of the volume tube could yield more accurate temperature compensation results. Additionally, we evaluated the measurement uncertainty of the device, finding that the relative expanded uncertainty of the volume measurement was only 0.012% (*k* = 2). Previously, the China National Institute of Metrology used the volumetric method to measure the pipe prover, and the measurement uncertainty reached 0.033%. Compared with this result, the measurement accuracy of the pipe prover measured by the dimensional method has been significantly improved. Thus, the proposed in situ measurement method effectively shortens the traceability chain and achieves a high level of accuracy in practical applications. An uncertainty analysis of the volumetric tube volume measured by the dimensional method was also performed, further affirming the method’s reliability.

## Figures and Tables

**Table 1:** Nominal volumes of pipe prover segments.

Pipe Segments	Volume (dm^3^)
P_1_	150.76
P_2_	150.80
P_3_	150.67
P_4_	150.55
P_1_–P_4_	602.78

**Table 2:** Φ313 mm ring gauge parameter.

Ring Gauge Position	Inside Diameter (mm)	Uncertainty *k* = 2 (mm)
Upper	312.9948	0.004
Middle	312.9950	0.004
Lower	312.9948	0.004

**Table 3:** Φ320 mm ring gauge parameters.

Ring Gauge Position	Inside Diameter (mm)	Uncertainty *k* = 2 (mm)
Upper	320.0002	0.004
Middle	320.0002	0.004
Lower	320.0002	0.004

**Table 4:** Fitting parameters.

Parameter	Value
*l*_1_	106.4200 mm
*l*_2_	106.8183 mm
*l*_3_	106.4932 mm
*θ*_12_	120.1452°
*θ*_23_	119.7563°
*θ*_31_	120.0985°
*r*_1_	0.0857 mm
*r*_2_	0.1152 mm
*r*_3_	0.4601 mm

**Table 5:** Pipe prover inner diameter measurements.

Pipe Segments	Temp (°C)	Inner Diameter of Pipe Segments (mm)
DP_1_	25.38	314.3501
DP_2_	24.28	314.2917
DP_3_	25.11	314.2097
DP_4_	24.12	314.1794

**Table 6:** Pipe prover length measurements.

Pipe Segments	Start Position (mm)	End Position (mm)	Revised Length (mm)
LP_1_	2038.4513	3982.0110	1943.5597
LP_2_	3990.5124	5931.1777	1940.6653
LP_3_	5923.0308	7866.9834	1943.9526
LP_4_	11,513.6269	13,456.1795	1942.5526

**Table 7:** Pipe prover volume calculations.

Pipe Segments	Volume (dm^3^)
P_1_	150.84
P_2_	150.58
P_3_	150.74
P_4_	150.60
P_1_–P_4_	602.74

**Table 8:** Pipe prover single section inner diameter.

Number	Inner Diameter Value (mm)	Number	Inner Diameter Value (mm)
1	314.1697	6	314.1671
2	314.1735	7	314.1677
3	314.1616	8	314.1696
4	314.1701	9	314.1757
5	314.1731	10	314.1730

**Table 9:** Uncertainty in inner diameter measurement.

Source	Error (μm)	Coverage Factor	Uncertainty (μm)	Proportion
Repeatability of the inner diameter measurement results *u*_D1_	4.05	$\sqrt{10}$	1.28	8.71%
Indication error of the inner diameter measurement *u*_D2_	1.85	$\sqrt{3}$	1.07	6.08%
Temperature compensation error of the standard ring gauge *u*_D3_	0.19	$\sqrt{3}$	0.11	0.06%
Standard ring gauge linear expansion coefficient error *u*_D4_	0.76	$\sqrt{3}$	0.44	1.03%
Standard ring gauge traceability *u*_D5_	4.00	2	2.00	21.26%
Inner diameter error of the measurement results caused by pipe roundness *u*_D6_	5.87	$\sqrt{3}$	3.39	61.08%
Pipe prover radial temperature compensation error *u*_D7_	0.14	$\sqrt{3}$	0.08	0.03%
Pipe prover radial linear expansion coefficient error *u*_D8_	0.55	$\sqrt{3}$	0.32	0.54%
Pipe prover pressure deformation error *u*_D9_	0.80	$\sqrt{3}$	0.46	1.12%
Pipe prover hydraulic pressure deformation error *u*_D10_	0.21	$\sqrt{3}$	0.12	0.08%

**Table 10:** Length of pipe segment measurement uncertainty.

Pipe Segment	Switch Uncertainty (μm)	Length Uncertainty (μm)
P_1_	11.94	17.32
	12.54	
P_2_	11.96	16.34
	11.14	
P_3_	12.08	18.52
	14.04	
P_4_	11.74	15.65
	10.34	

**Table 11:** Uncertainty component in length measurement.

Source	P_1_ (μm)		P_2_ (μm)		P_3_ (μm)		P_4_ (μm)	
	Error	Uncertainty	Error	Uncertainty	Error	Uncertainty	Error	Uncertainty
Repeatability *u*_L1_	17.32	17.32	16.34	16.34	18.52	18.52	15.64	15.64
Measurement error *u*_L2_	2.59	1.50	3.59	2.07	4.70	2.71	5.81	3.36
Signal delay *u*_L3_	1	0.58	1	0.58	1	0.58	1	0.58
Temp variation *u*_L4_	3.36	1.94	3.36	1.94	3.36	1.94	3.36	1.94
Linear expansion coefficient *u*_L5_	13.44	7.76	13.43	7.75	13.45	7.77	13.44	7.76

**Table 12:** Length measurement uncertainty.

Pipe Segment	Length Uncertainty (μm)
P_1_	19.15
P_2_	18.32
P_3_	20.37
P_4_	17.89

**Table 13:** Sensitivity coefficient.

Pipe Segment	*c*_L_ (mm^2^)	*c*_D_ (mm^2^)
P_1_	77,609.8934	959,690.8745
P_2_	77,581.0594	958,083.6517
P_3_	77,540.5822	959,456.1617
P_4_	77,525.6281	958,672.7224

## Data Availability

Data are available upon request.
